# Carvacrol and HP-β-Cyclodextrin Complexes: Extensive Characterization and Potential Cytotoxic Effect in Human Colorectal Carcinoma Cells

**DOI:** 10.3390/pharmaceutics14122638

**Published:** 2022-11-29

**Authors:** María Isabel Rodríguez-López, María Teresa Mercader-Ros, Alfonso Pérez-Garrido, Horacio Pérez-Sánchez, José Antonio Pellicer, Carmen Lucas-Abellán, Silvia Montoro-García, María Josefa Yáñez-Gascón, Ángel Gil-Izquierdo, Estrella Núñez-Delicado, José Antonio Gabaldón

**Affiliations:** 1 Molecular Recognition and Encapsulation Research Group (REM), Health Sciences Department, Universidad Católica de Murcia (UCAM), Campus de los Jerónimos 135, E-30107 Guadalupe, Spain; 2 Bioinformatics and High Performance Computing Group (BIO-HPC), Dpto. del Grado en Informática, Universidad Católica de Murcia (UCAM), Campus de los Jerónimos 135, E-30107 Guadalupe, Spain; 3 Cátedra de Riesgo Cardiovascular y Departamento de Nutrición, Facultad de Ciencias de la Salud, UCAM, Universidad Católica de Murcia (UCAM), Campus de los Jerónimos 135, E-30107 Guadalupe, Spain; 4 Research Group on Quality, Safety and Bioactivity of Plant Foods, Department of Food Science and Technology, CEBAS-CSIC, University Campus of Espinardo—Edif. 25, E-30100 Espinardo, Spain

**Keywords:** carvacrol, HP-β-cyclodextrins, solid complexes, microwave irradiation, chemical characterization, cell viability

## Abstract

The aim of this study was to obtain solid carvacrol-cyclodextrin (CD) complexes for use in the pharmaceutical industry. To this end, the complexation of carvacrol at different pH values was studied in detail, to determine the type of CD and the reaction environment that supported the highest amount of encapsulated carvacrol. Evidence of the capability of hydroxypropyl-β-cyclodextrins (HP-β-CD) to form inclusion complexes with carvacrol (K_C_ = 5042 ± 176 L mol^−1^) and more high complexation efficiency (2.824) was demonstrated for HP-β-CDs using two different energy sources, ultrasound (US) (K_C_ = 8129 ± 194 L mol^−1^ 24 h) and microwave irradiation (MWI) (K_C_ = 6909 ± 161 L mol^−1^), followed by spraying the resulting solution in a spray dryer. To confirm complex formation, the complexes were characterized using various instrumental methods to corroborate the carvacrol incorporation into the hydrophobic cavity of HP-β-CD. The obtained carvacrol solid complexes were analyzed by 1H nuclear magnetic resonance (^1^H-NMR) and 2D nuclear magnetic resonance (ROSEY), differential scanning calorimetry (DSC), thermogravimetric analysis (TG) and Fourier transform infrared spectroscopy (FTIR) characterization. The structures of the resulting complexes were also characterized by molecular modeling. Furthermore, 1 mM HP-β-CD-carvacrol complex has been shown to reduce cell proliferation in HCT-116 colorectal cancer cells by 43%, much more than in a healthy lung fibroblast MRC-5 cell line (11%).

## 1. Introduction

Recently, the demand for natural substances that can be used as preservatives or new drugs in the food or pharmaceutical industries has increased significantly, mainly due to the negative perceptions of chemically synthesized compounds by consumers. Within this group, essential oils (EOs) and their constituents offer viable short-term solutions.

An EO is a very complex natural mixture that can contain anywhere from 20 to 60 different concentrations of ingredients. All the mixtures in this study showed high concentrations (20–70%) of the major component. Those components that appear in greater proportion are those that determine their biological properties [1], and among these majority groups, terpenes, on the one hand, and on the other, the aromatic components are all characterized by their low molecular weight and high volatility [2]. However, this composition may change considerably, depending on the influence of environmental factors such as humidity, soil type, temperature and collection period, conditioning at the time of obtaining the oil, and the richness of some compounds, thereby modifying their biological properties [3,4,5,6]. EOs have a wide range of biological activities, such as antifungal, antimutagenic, anticancer, antiviral, antioxidant, antidiabetic, and anti-inflammatory activity [7,8,9,10,11,12,13]. However, their use in the food or pharmaceutical industries has been limited. This is because they present action-sensitive hydrophobic compounds that are sensitive to external factors, such as oxygen, ultraviolet light, and temperature, and undergo structural changes catalyzed by various oxidases. In addition, their intense flavor allows them to change their organoleptic characteristics when added to food products, leading to consumer rejection; they may even cause contact or ingestion allergy in fragrances at the concentrations commonly used in perfumery, which may cause the emergence of pre-existing defects in their formulation and performance in various industries (food, cosmetic, chemical, or pharmaceutical, etc.).

Therefore, given the advantages of EOs, it would be interesting to develop strategies to overcome the inherent drawbacks of their physicochemical properties, which have so far limited their widespread use. To overcome these limitations, several researchers have found a solution in the use of different encapsulation techniques, such as complex coacervation with chitosan and cashew gum, spray-drying with gum Arabic [14], the use of maltodextrin and modified starch [15], solutions supercritical to surfactants with liposomes [16], and ionic gelation with chitosan [17].

Among the possible alternative solutions, microencapsulation by means of cyclodextrins (CDs) was proposed. CDs are cyclic carbohydrates that are capable of forming inclusion complexes with a wide variety of substances. Encapsulation through CDs provides various advantages, as it increases solubility, protects the encapsulated guests from a harmful environment, prevents interaction with the components of the food matrix, creates a controlled release system, reduces the formation of off-flavors, and preserves the true aroma of the food characterization [18,19,20]; therefore, CDs have found an important place in the food and pharmaceutical industries.

As previously mentioned, EOs have a large number of different components, but their biological properties are attributed to the main components. Therefore, carvacrol, a major component of oregano EO, has been selected in this study because CDs are not selective when incorporating molecules in their interior so that the use of pure molecules so as to be able to identify the effect of the complexation is desirable.

The inclusion complexes that are formed between CD and its guest compound need to be characterized using different analytical techniques. In different studies, the complexes formed by β-CDs and carvacrol have been characterized using different techniques, such as DSC (differential scanning calorimetry) or TG (thermal gravimetric analysis), as they provide a rapid and reliable evaluation of the interactions between CD and carvacrol [21,22], via X-ray crystallography, as it provides the most accurate and absolute structural information of the complex in the crystalline state [23], or by scanning electron microscopy (SEM), which provides us with information on the complex’s morphology [24]. However, the inclusion complexes formed by carvacrol and HP-β-CDs have not yet been studied in detail, as in the case of β-CDs [25].

Therefore, the objective of the present work was to achieve the complexation of carvacrol in CDs to overcome the drawbacks described and enable its possible industrial use. Complexation with different types of cyclodextrins will thus be carried out, to select the type that obtains the best complexation constant, and to optimize a method to prepare solid complexes of carvacrol, evidencing its inclusion into the CDs hydrophobic cavity by ^1^H-NMR, DSC), TG, and FTIR techniques. For this study, its geometry and energetically favorable conformation were explored by applying scanning electron microscopy (SEM) and molecular docking. Finally, its antiproliferative effect and cytotoxicity against HCT-116 colorectal carcinoma cells were evaluated in vitro.

## 2. Materials and Methods

### 2.1. Materials

Carvacrol at 99.5% purity and methanol-d_4_ were purchased from Sigma (Sigma-Aldrich, Madrid, Spain). The α-, β-, HP-β-CDs were obtained from Henan Puertai Animal Medicine Co., Ltd. (Zhengzhou, China). All the other chemicals used were of analytical grade.

### 2.2. Study of Solubility at Different pH Levels

The solubility studies of carvacrol, in the presence of CDs, were performed according to the method described by Higuchi and Connors in 1965, with slight modifications [26]. For this purpose, aqueous solutions of increasing concentrations of α-, β-, and HP-β-CDs were created, until they reached a concentration of 100 mM, while in the case of β-CDs, they reached up to 15 mM, which is its solubility limit in water, at a total volume of 5 mL. For an evaluation of the pH effect in the process of complex formation, these solutions were prepared in different buffer solutions: 100 mM sodium acetate buffer, pH 3.5 and 5.5; 100 mM sodium phosphate buffer, pH 6.5 and 7.0; and 100 mM sodium borate buffer, pH 8.5. To each solution, a saturating amount of the carvacrol under analysis was added, then the solutions were kept in an ultrasonic bath for 60 min in the dark at 25 °C until equilibrium was reached. After this point, the solutions were filtered through 0.45 μm nylon to remove the excess carvacrol.

#### Quantification of Essential Carvacrol by GC-MS Analysis

To quantify the amount of monoterpene in each filtrate, the complexes were disrupted by adding 80% ethanol. Each solution was then introduced into a gas chromatograph, coupled to a mass spectrometer (Shimadzu QP 2010) equipped with an Slb-5 ms (30 m × 0.25 mm × 0.25 mm) capillary column. The operating conditions are as follows: the starting temperature is 70 °C, the temperature is increased at a rate of 4 °C/min until it reaches 160 °C and is then increased to 280 °C at a rate of 30 °C/min, then held there for 6 min; the inlet temperature is 250 °C, the injection type is a 1:20 split mode, with helium used as the carrier gas. Component identification is based on the relative elution times and comparison of the mass spectra of each peak against a database containing the system.

Once the concentrations of carvacrol in each sample were quantified, the values obtained were plotted against the concentrations of CDs. For the calculation of K_C_, the slope and intercept of the phase diagrams related to the solubility studies were determined, according to Equation (1) [26]:

(1) $$K_{C}=\frac{\mathrm{Slope}}{S_{0}\;\left(1-\mathrm{Slope}\right)}$$

where S_0_ is the solubility of carvacrol at 25 °C, in the absence of cyclodextrins, and Slope is the gradient of the phase solubility diagrams.

To determine the complexation efficiency (CE) of carvacrol, Equation (2) was used [27]:

(2) $$\mathrm{CE}=\frac{\left[\mathrm{carvacrol}-\mathrm{CDs}\right]}{\left[\mathrm{CD}\right]}=S_{0}\times {\;K}_{C}.$$

The molar ratio (MR) of carvacrol:CDs was calculated using the CE values and Equation (3) [28]:

(3) $$\mathrm{carvacrol}:\mathrm{CDs}=1:\left(1+\frac{1}{\mathrm{CE}}\right).$$

### 2.3. Preparation of the Solid Complexes of Essential Carvacrol

#### 2.3.1. Ultrasound (US)

The solubility method was developed from that used by Higuchi and Connors, with minor modifications [26]. We added an excess of carvacrol to an aqueous solution that increased the concentration to 100 mM HP-β-CD, in 100 mL of water, at 25 °C. The samples were kept in an ultrasound bath (P-Selecta Ultrasounds, Barcelona, Spain) for 60 min to equilibrate. The aqueous solutions were filtered through 0.45 µm nylon to remove the excess free carvacrol. To determine the amount of carvacrol complexed, samples were diluted in ethanol-water at 80% and analyzed via gas chromatography–mass spectrometry (CG–MS).

#### 2.3.2. Complexation Process Using Microwave Irradiation (MWI)

The calculation of the complexation constant (K_C_) between carvacrol and HP-β-CDs was carried out by the addition of different concentrations of HP-β-CDs to 100 mL of water, maintaining a 1:1 molar ratio. Aqueous HP-β-CD solutions (0–100 mM) were irradiated with microwaves for 30 s at 10 s intervals until the dissolution reached 70 °C. The carvacrol was then added to each sample and irradiated again for 30 s at the same intervals (10 s) until the samples reached 70 °C. Subsequently, the samples were shaken and held for 12 h in scintillation vials, sealed with parafilm, and protected from light.

Then, we repeated the above procedure by irradiating the sample again until it reached 70 °C. The solution was then filtered through 0.45 μm nylon to remove the excess carvacrol. Finally, the concentration of monoterpene was determined via CG-MS.

#### 2.3.3. Spray Drying

For each complex (carvacrol-HP-β-CD), the obtained aqueous solutions were subjected to the atomization process via two procedures (MWI and US) to obtain solid complexes. A Mini Spray Dryer Büchi B290 (Flavila, Flawil, Switzerland) was used for this purpose. The following drying process parameters were determined to be optimal: inlet temperature 170 ± 2 °C, outlet temperature 68 ± 2 °C, flow rate 5 mL/min, air pressure 3.2 bar, rotation speed 30,000 rpm, and nozzle diameter 1.5 mm.

#### 2.3.4. Determination of Yield (Y), Encapsulation Efficiency (EE), and Drug Loading (DL)

To determine the spray process (AP) performance of each monoterpene-HP-β-CD complex, the weight (in grams) of the solid complexes obtained after spray-drying was determined from the weight of the corresponding solution complex in grams, in accordance with an earlier study [29]:

(4) $$\mathrm{Yield}\;\left(\%\right)=\frac{\mathrm{Weight}\;\mathrm{of}\;\mathrm{solid}\;\mathrm{dispersion}}{\mathrm{Total}\;\mathrm{weight}\;\mathrm{of}\;\mathrm{CDs}\;\mathrm{and}\;\mathrm{carvacrol}}\times 100.$$

While the yield (Y) gives an idea regarding the amount of solids (CD and carvacrol) in solution and in solid form, the encapsulation efficiency (EE) refers to the ratio of monoterpenes in the solid form to the content in the solution (excluding CD). The calculation is described in an earlier study [30] and follows Equation (5):

(5) $$\mathrm{EE}\;\left(\%\right)=\frac{\mathrm{Amount}\;\mathrm{of}\;\mathrm{active}\;\mathrm{compound}\;\mathrm{entrapped}}{\mathrm{Initial}\;\mathrm{amount}\;\mathrm{active}\;\mathrm{compound}\;}\times 100$$

where the “amount of active compound entrapped” is the compound present in the inclusion complex particle, and the “initial amount active compound” indicates the compound that was initially used to manufacture the inclusion complex particle. In addition, the active matter loading (AML) or drug loading (DL) values were determined by Equation (6):

(6) $$\mathrm{DL}\;\left(\%\right)=\frac{\mathrm{Amount}\;\mathrm{of}\;\mathrm{active}\;\mathrm{compound}\;\mathrm{entrapped}}{\mathrm{Amount}\;\mathrm{of}\;\mathrm{particles}\;\mathrm{produced}}\times 100$$

### 2.4. Characterization of Solids Complexes of Essential Carvacrol

#### 2.4.1. NMR Study of the Supramolecular Structure of Carvacrol/HP-β-CDs Complex

NMR experiments were performed at 298 °K on a Bruker Avance 600 Mhz spectrometer, fitted with a 5-millimeter TXI probe. Because of the very low solubility of carvacrol in water, spectra were also acquired in a solution of methanol-carvacrol, HP-β-CDs, and carvacrol/HP-β-CDs complex. First, 10 mg of samples were individually dissolved in 0.60 mL of methanol-d_4_. Then, the characterization of carvacrol and HP-β-CDs was performed by means of a ^1^H (600.13 MHz) experiment. The chemical shifts relative to a methanol-d_4_ internal standard (δ = 3.343) were expressed in parts per million (ppm). A two-dimensional rotational frame nuclear Overhauser-effect spectroscopy (2D ROESY) experiment was performed at 600.13 Mhz, using the standard Bruker pulse program, roesygpph [31].

#### 2.4.2. Molecular Docking Simulations

The molecular structures used in this study were erected manually using AutoDock tools [32], or were deduced from the experimental data. The structure of β-CD was taken from the crystal structure stored in the Protein Data Bank (PDB), using the code 3CGT. The structure of the HP-β-CD model was constructed by adding the hydroxylpropyl groups to the β-CD model. Molecular docking calculations were performed, using the AutoDockVina default parameters [33]. The hydroxylpropyl groups of HP-β-CD were found to be particularly flexible during the docking simulations. Graphical representations of the docking results were prepared using PyMOL (Molecular Graphics System, Version 1.3, Schrödinger, LLC, New York, NY, USA).

#### 2.4.3. Differential Scanning Calorimetry and Thermogravimetric Analysis

Differential scanning calorimetry (DSC) was used to study the formation of complexes between carvacrol and HP-β-CD. Analysis was performed using a DSC Q100 (TA Instruments, Cerdanyola del Valles, Spain) with a scanning rate of 10 °C/min, from 25 °C to 300 °C, under a nitrogen atmosphere. Samples of free carvacrol, HP-β-CD, and carvacrol complex (4–5 mg) were weighed to the nearest 0.1 mg, transferred to aluminum capsules, and sealed.

Thermogravimetric analysis (TG) characterizes those materials that show weight loss or gain due to decomposition, oxidation, or dehydration; therefore, TG is used for the characterization of inclusion complexes. First, 4–5 mg of the sample was weighed in aluminum capsules for the experiment, which was carried out in a Hi-Res TGA 2950 thermogravimetric analysis machine (TA Instruments, Cerdanyola del Valles, Spain). Then, the samples were subjected to a heating ramp from room temperature to 300 °C, at a rate of 10 °C/min, under a nitrogen atmosphere.

#### 2.4.4. Fourier Transform Infrared Spectroscopy (FTIR)

Fourier transform infrared spectroscopy was used to study the changes in the chemical structures of free carvacrol and carvacrol complexes. For the assay, 10 mg of samples were analyzed in a Varian FT-IR 670 Spectrophotometer (Varian Inc., Palo Alto, CA, USA). The spectral range was 500–4000 cm^−1^, using 128 scans and a resolution of 0.10 cm^−^^1^ in ATR mode.

#### 2.4.5. Scanning Electron Microscopy (SEM)

Dried complexes were mounted on aluminum stubs, coated with a thin layer of gold, and visualized with a JEOL Model JSM-6100 scanning electron microscope at an accelerated voltage of 25 Kev.

### 2.5. Cell Culture

Colorectal adenocarcinoma (HCT-116) and non-tumoral fetal lung fibroblast (MRC5) cell lines were obtained from the American Type Culture Collection (ATCC, Rockville, MD, USA). The cell lines were cultivated in high glucose Dulbecco’s modified Eagle’s medium (DMEM) and Eagle’s minimum essential medium (EMEM), respectively, containing 10% heat-inactivated fetal bovine serum (FBS), 2 mM glutamine, 50 U/mL penicillin and 50 µg/mL streptomycin (Sigma-Aldrich Chemical Co., St. Louis, MO, USA) in an atmosphere of 5% CO_2_ and 95% humidified air at 37 °C. The subculture was complete when 90% confluence was reached. Fibroblasts for up to 15 doublings were used for the experiments.

#### 2.5.1. Cell Viability Assay

Exponentially growing cells were plated in triplicate in flat-bottomed 96-well plates (Nunc, Roskilde, Denmark) at 1500 cells/well for HCT-116 and 4000 cells/well for MRC5. The following day, compounds were added in serial dilutions, from 50 µM to 1000 µM. Control wells contained compound-free medium plus 0.1% dimethyl sulfoxide (DMSO). The plates were incubated for 3 days in a humidified 5% CO_2_ incubator and cell viability was determined. Tetrazolium, dissolved in phosphate-buffered saline (PBS), pH 7.2, at 1.9 mg/mL (30 µL per well) was added to the cells After 4 h of incubation at 37 °C, the medium was aspirated. The formazan crystals were dissolved in 200 µL DMSO for 30 min and the absorbance was read at 570 nm in a microtiter plate reader. The results were calculated as:

(7) $$\mathrm{cell}\;\mathrm{viability}\;\left(\%\right)=\frac{\mathrm{average}\;O.D\;\mathrm{of}\;\mathrm{wells}}{\mathrm{average}\;O.D\;\mathrm{of}\;\mathrm{control}\;\mathrm{wells}}.$$

#### 2.5.2. In Vitro Release

Carvacrol release from the free and complexed carvacrol was monitored by chromatography (see Section 2.2). Briefly, 1 × 10^5^ HCT-116 cells were grown for up to 7 days in DMEM, supplemented with 300 µM free or complexed HP-β-CD-carvacrol, at 37 °C. At each appropriate time point (days 1–7), 1 mL aliquots were withdrawn and replaced with an equal volume of fresh medium. All determinations were made in triplicate.

### 2.6. Statistics

Data are presented as the mean ± standard deviation (SD). For the in vitro experiments, a one-way analysis of variance (ANOVA), followed by a Tukey post hoc test, was performed to compare each group. Differences were considered significant with an error probability of *p* < 0.05. SPSS 18.0 software was used for the other statistical analyses (SPSS, Inc., Chicago, IL, USA).

## 3. Results and Discussion

### 3.1. Formation of Inclusion Complexes at Different pHs

**Figure 1:**
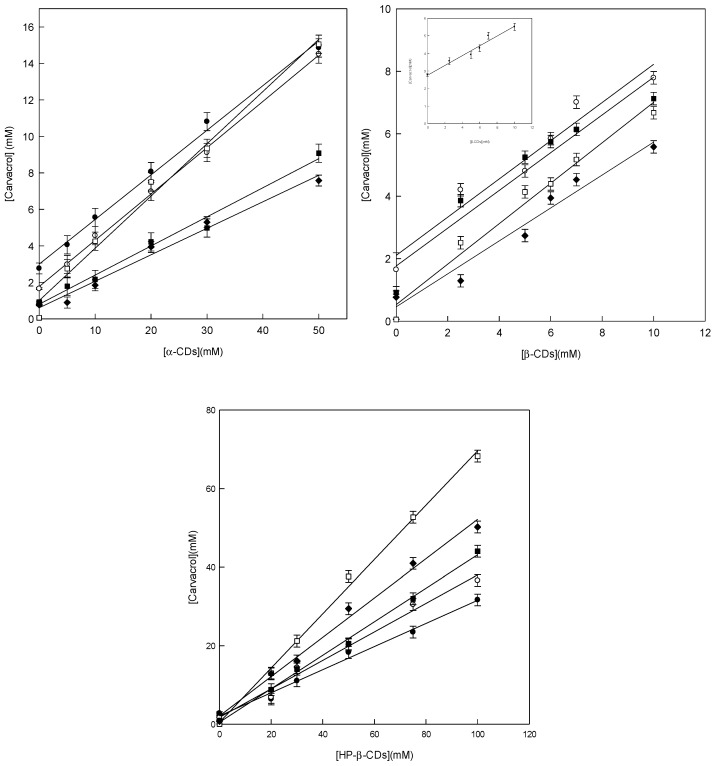
Phase diagrams of carvacrol with α-, β-, and HP-β-CDs at different pH values: pH 3.5 (●), pH 5.5 (○), pH 6.5 (■), pH 7.0 (□), and pH 8.5 (♦), at 25 °C. Values represent the means of triplicate determination. Inset pH 3.5 (●) at 25 °C.

The solubility method was used to calculate the K_C_ value between carvacrol and the different types of CDs, using the method described by Higuchi and Connors in 1965 [26], with slight modifications. The carvacrol phase diagrams showed a linear trend for the three types of CDs; as the concentration of CDs increased, the concentration of carvacrol (Type A_L_) increased, indicating that the stoichiometry of the inclusion complexes that were formed was 1:1 in all cases (Figure 1).

As shown in Table 1, the K_C_ values obtained by the solubility method increased as the pH was close to 7.0; this is because the solubility (S_0_) of carvacrol varies according to the pH of the medium (Table 1). In fact, it dissolves better at an acidic pH (S_0_ = 2.76) than at 7.0, decreasing its solubility by 80%. However, at an alkaline pH (8.5), it rises again to 0.76, reaching a solubility close to that obtained at a pH of 6.5 (S_0_ = 0.92).

**Figure 2:**
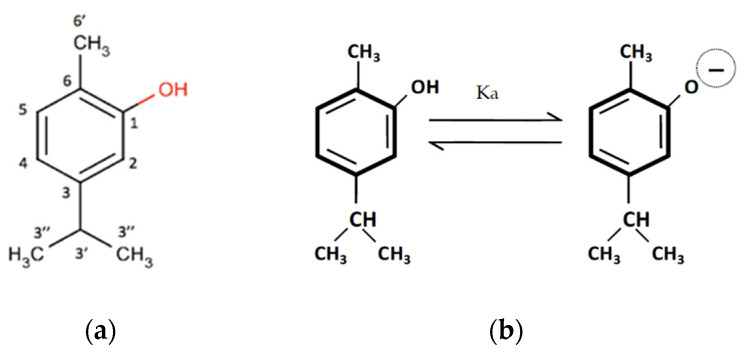
(**a**) Structure of carvacrol with H-atoms. (**b**) Carvacrol dissociation equilibrium.

The activity of carvacrol is conditioned by its structure. It behaves as a weak acid (pKa = 10.38), depending on the pH of the medium. Therefore, it is possible to modify its degree of dissociation, based on the pH and, therefore, its solubility. In an aqueous solution at a basic pH, it comes into contact with alkaline hydroxides; being more acidic than water, it reacts with them to form salts or phenoxide ions that are more stable than carvacrol itself, due to the off-sloping of the negative charge through the aromatic ring (Figure 2).

When carvacrol is entrapped in the CD cavity, the entropy of the system increases, reducing the overall free energy of this linkage, which causes an increase in the stability of the complex. It should be taken into account that the factors involved in complex formation are the size of the CD and the complexed molecule and their polarity.

Therefore, the protonation of carvacrol will determine the stability of the complexes. In fact, in this study, it has been verified that for the three types of CDs tested, a large increase in the stability of the complexes obtained at pH 7.0 is observed. This is significantly different from the results achieved by the complexes formed by its phenoxide ion, which, being more soluble than its non-dissociated form, is less favored for inclusion in the interior of the CDs.

Another factor that affects the value of K_C_ is the size of the cavity of the CDs. Thus, when evaluating the value of the native CDs (Table 1), it can be seen that this is higher for β-CDs, regardless of the pH value tested. These differences in the value of K_C_ of α-CDs and β-CDs are due to the size of the cavity of the CDs, this being higher in the case of β-CDs. For this reason, the K_C_ value was 8 times higher than that determined with α-CD (400 ± 56).

Therefore, the K_C_ value between α-CDs and β-CDs can be explained by the differences in the size of the CD cavity, but if β-CDs, with their modified HP-β-CDs, are compared, one might think that both should have the same K_C_ value (3466 ± 115 and 5042 ± 176, respectively), since they have the same diameter; however, when looking at the values obtained, it is clear that this is not the case.

A possible explanation for this fact is that HP-β-CD forms a more stable complex with carvacrol than that obtained with its native (β-CD), since the hydroxypropyl groups present in the modified complex break the H-bridges near the CD cavity, making it more accessible for the molecules of carvacrol to the apolar cone, causing an increase in the solubility of carvacrol.

In addition, it should be taken into account that the hydroxypropyl groups promote conformational distortion so as to better accommodate the host, resulting in the opening of the cyclodextrin cavity, significantly modifying the size with respect to the native one (similar to the dilation of the snake’s mouth (DSM effect)); in consequence, the carvacrol molecule completely enters the internal cavity of the HP-β-CDs, whereas, in β-CDs, only part of the molecule has access to the hydrophobic cavity. These results are consistent with those obtained for thymol [34], kaempferol, and p-coumaric acid, which have cyclic structures, or citral and linalool, which have a non-cyclic structure [35,36].

It is important to determine the complexation efficiency (CE) when CD saturation studies are performed. Besides Kc, this factor takes into account the solubility of the encapsulated compound (S_0_) at different pH values. For complexes with 1:1 stoichiometry, the CE can be calculated from the slope of the phase diagram (Equation (2)), since it considers both the solubility of carvacrol and K_C_. The CE value obtained for carvacrol, with the different types of CDs at different pH values, is summarized in Table 1.

The solubility product varies according to the pH of the medium. As can be seen in Table 1, carvacrol can be converted from its neutral form to its protonated form, causing a significant variation in its solubility. In addition, if the CE values are compared, it can be seen that the highest values are obtained for both β-CDs (1.941) and their modified HP-β-CDs (2.824) at a neutral pH, the values being the highest for HP-β-CDs. Similar results were previously described for isoeugenol with HP-β-CDs, RAMEB (randomly methylated-β-cyclodextrin), and CRYSMEB (methyl-beta-cyclodextrin) (1.82, 1.73, and 1.26, respectively), and p-coumaric acid with HP-β-CDs, RAMEB, and CRYSMEB (2.30, 2.57, and 1.89, respectively) [37]. CE was then used to calculate the molar ratio (MR) of carvacrol:CD using Equation (3).

As shown in Table 1, the best MR (1:1) matches with the highest CE value. This result was obtained for HP-β-CDs at pHs 6.5 and 7.0 (1:1 in all cases), and for β-CDs at pHs 5.5 and 7.0 (Table 1). This 1:1 value indicated that almost all the CDs in the solution formed soluble complexes with carvacrol. These results agree with those obtained with linalool, where the highest CE values correspond to an MR ratio of 1:1 [36].

### 3.2. Preparation of the Solids Complexes of Essential Carvacrol with HP-β-CDs

Once the ability of HP-β-CDs to form complexes with carvacrol was checked, they were selected to carry out the formation of inclusion complexes at a neutral pH, later atomizing the solutions in the Spray Dryer to obtain complexes in a solid state since this facilitates their handling and conservation.

**Figure 3:**
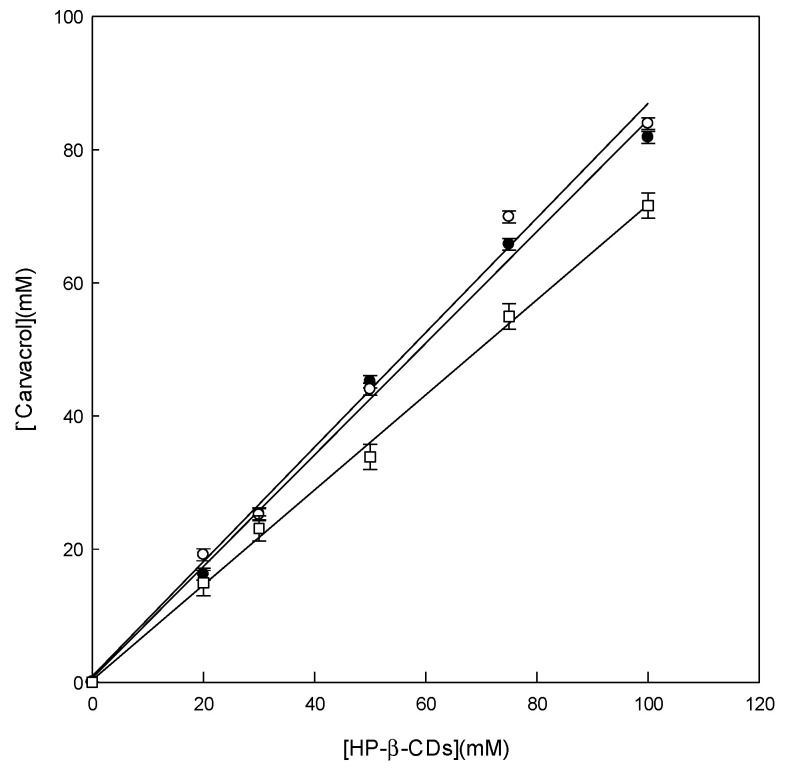
Carvacrol complexes with HP-β-CD at pH 7.0, performed at 24 h (●) and 48 h by microwave irradiation (MWI) (○) and ultrasound (US) (□). The experiments were performed in triplicate.

Two different energy sources were used to form the solid complexes: MWI [38] and US. Figure 3 shows that the stoichiometry of the complexes formed is 1:1 for both MWI (24 and 48 h) and US because when the concentration of HP-β-CDs increased, the concentration of carvacrol simultaneously increased.

The constant values (K_C_) for MWI are slightly higher with less contact time (8129 ± 194 L mol^−^^1^, at 24 h) than those determined after lengthening the process by one more day (6811 ± 107 L mol^−^^1^, at 48 h). It was also determined that the increase in solubility of carvacrol is 151.8 and 155.6 times that at 24 h and 48 h, respectively, values that are very similar to each other. Thus, to optimize the formation of inclusion complexes with carvacrol by this method, the product obtained at 24 h was selected, since a higher K_C_ value ensures the formation of more stable complexes. In addition, the consequent saving of energy and time in the preparation of the inclusion complexes foresees a simpler technology for a future industrial application. The comparison of the K_C_ value obtained by MWI (8129 ± 194 L mol^−^^1^, at 24 h) and by US (6909 ± 161 L mol^−^^1^) showed quantitative discrepancies. The K_C_ value is different because the S_0,_ in the case of MWI (0.35 mmol L^−^^1^), is slightly lower than that achieved by US (0.56 mmol L^−^^1^). This could be explained by the fact that in the case of MWI, the molecular heating phenomenon is not sufficient to cause the disintegration of the microdroplets of carvacrol oil. On the other hand, for US, the disintegration of the drops is favored by molecular movement. These results are in concordance with those obtained when complexing carvacrol with HP-β-CDs by MWI irradiation [36]. If the increase in the solubility of carvacrol by MWI irradiation and by the US method is compared, it can be verified that this is 151.8 and 132.9 times, respectively, so this increase is higher for the MWI irradiation. In this instance, soluble complexes prepared with different concentrations of HP-β-CDs were spray-dried to obtain the solid-state complexes. This drying method is the most common and economically viable method for the microencapsulation of powdered ingredients for food applications [34,38].

**Figure 4:**
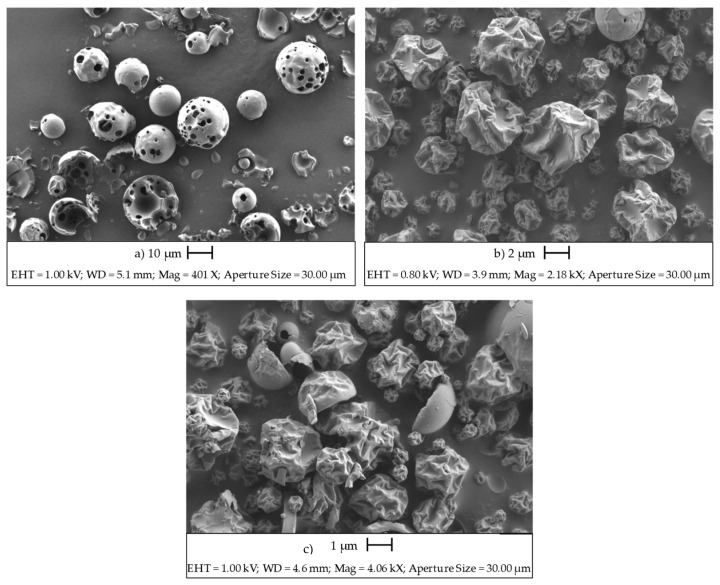
Micrographies of HP-β-CDs (**a**), MWI complexes (**b**), and US complexes (**c**).

According to Dufour et al. [39], the shape of the particles after the drying process is influenced by the solids content in the solution and, therefore, the time it takes the solute to reach the surface of the drop. Other parameters are also involved, such as the inlet temperature, the spray rate, and the air nozzle of the equipment, which affect the rate of evaporation. Thus, the wrinkled form of the particles of the complex, formed by carvacrol-HP-β-CD (Figure 4), is due to the fact that the evaporation rate on the droplet surface is faster than the diffusion of the dissolved components toward the center, forming a shell around the droplets [40].

As can be seen in photomicrographs b and c (Figure 4), the outer surfaces have continuous walls without cracks, which significantly affects the loss of volatile compounds. In the literature, similar morphologies are observed in the microencapsulation of lemongrass oil [41], ginger essential oil [42], and thyme essential oil [43], wherein microparticles with similar external globular morphology also appear as a result of the rapid evaporation of the water during the spray-drying process.

**Figure 5:**
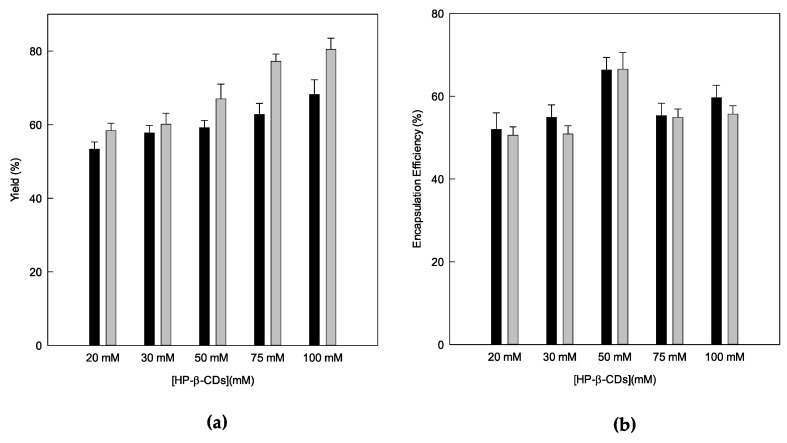
(**a**) Drying process yield (g kg^−1^) and (**b**) encapsulation efficiency (g kg^−1^) of carvacrol-HP-β-CDs complexes, prepared via US (grey bars) and MWI (black bars). The experiments were performed in triplicate.

Subsequently, the drying performance calculation was performed using Equations (4) and (5), preparing the complexes via MWI irradiation and US. The powder remaining inside the equipment was discarded as being of a negligible amount. As shown in Figure 5, the yield was greater than 50% in all cases, so it can be considered that a good yield was obtained for both methods. It is important to note that as the concentration of HP-β-CD increased, a higher yield was obtained for both the MWI and US, reaching the maximum yield at 100 mM via the solubility method (80.48%).

The difference between the two methods may be due to the fact that in the same proportion of CDs, there is more carvacrol in solution in the MWI than in US, which means that there is a greater amount of solids in the mixture; this increases the viscosity of the solution [44], which causes the solids to be in contact with the wall of the chamber, being able to adhere to it, and decrease the performance of the process [44]. In addition, it must be considered that the quantities that are introduced in the equipment are small (a total volume of 100 mL). For this reason, losses can always take place, since, on a laboratory scale, it is difficult to reach yields higher than 80% [45].

It is interesting to note that the spray-drying process was chosen to be scaled at an industrial level, since it is a fast and effective process, and it is also proven that increasing the drying volume can increase the performance of the process. This trend was observed in the study carried out by Eguinoa [46] involving eugenol, complexed with β-CDs.

Next, both the carvacrol’s losses and its concentration in the solid complexes after the drying treatment were calculated (Figure 5b). The EE is always higher for the MWI since it ranged from 51.96 to 66.37 g/100 g; however, for the US, it was from 50.86 to 66.55 g/100 g. One of the main reasons for this may be that in the MWI, the CDs reach an instantaneous state of resonance, thus favoring the exit of water molecules from the interior and the inclusion of carvacrol [47].

In order to corroborate the results obtained in the EE, the drug loading (DL) of carvacrol was calculated according to Equation (6), which relates to the amount of active material encapsulated with the grams of powder obtained via the Spray Dryer. As can be seen in Table 2, as with EE, the higher values are obtained for the MWI, ranging from 4.33 to 6.48 g/100 g, and, in the US technique, from 2.90 to 3.64 g/100 g, indicating that the amount of inclusion complexes formed by the MWI were higher than those obtained by the solubility method, in terms of uniformity. These results are similar to those obtained by Tao et al. [48] for carvacrol encapsulated in β-CDs.

It should be noted that a higher drying performance does not imply a high level of inclusion complexes in the powder, since active inclusion complexes and empty CDs may be present in the sample.

According to the previous results analyzed, MWI irradiation is the most appropriate method for the formation of the inclusion complexes of carvacrol and HP-β-CDs because it shows a higher value of K_C_ and, therefore, a higher EE, as well as a higher loading of active substances.

One desirable property of encapsulated monoterpenes is to be cost-effective as well as to endure long periods of storage before being used; therefore, it is not only important to determine the amount of the compound at the initial moment but also the amount retained during storage. In order to establish the stability of the solid complexes during storage, the spray-dried samples were selected with a concentration of 50 mM HP-β-CDs. Samples were stored for 17 months in sealed plastic containers and stored at 25 °C, with others at 8 °C.

**Figure 6:**
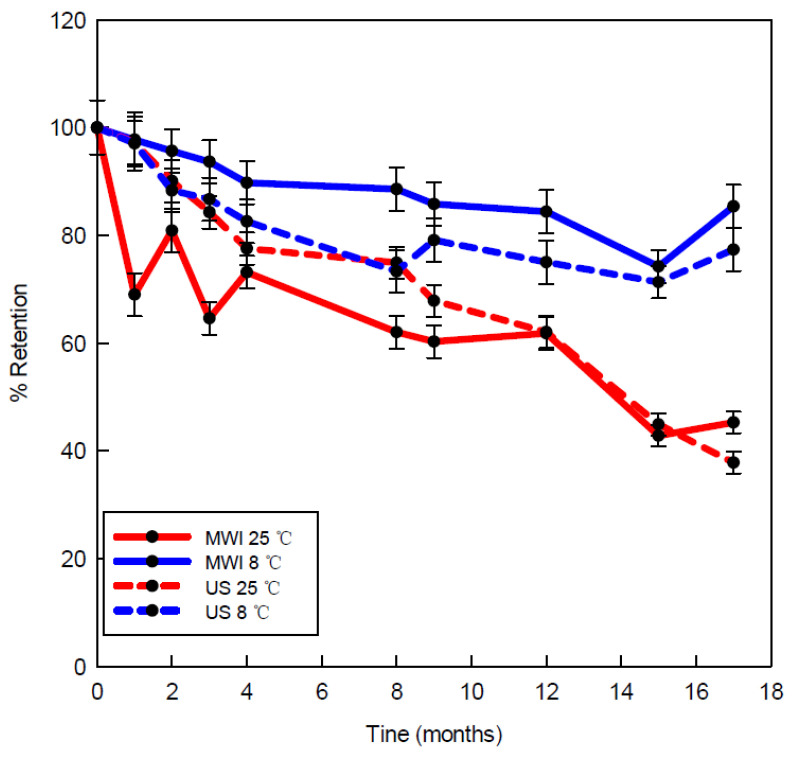
Stability of the solid complexes during storage. MWI at 25 °C (red line) and 8 °C (blue line), with US complexes at 25 °C (red dashed line) and 8 °C (blue dashed line).

As can be seen in Figure 6, the powder obtained via MWI and held in 17 months of storage has a higher rate of retention of encapsulated carvacrol at 8 °C, ranging from 100 to 74.23%, while at 25 °C, it ranged from 100 to 45.10%. During storage, the percentage of retention of carvacrol can be affected by both temperature and humidity since at 25 °C, with a relative humidity (RH) of 50%, there was a release in the content of CDs, while at 8 °C, with an RH of 19%, they remained within it for longer. This may be due to the fact that HP-β-CD has a higher affinity for water molecules as the RH increases because of the number of hydroxyl groups present on its surface, which makes it possible for it to release the complexed compounds [49].

### 3.3. NMR Study of the Supramolecular Structure of the Carvacrol/HP-β-CDs Complex

The formation of the inclusion complexes of carvacrol-HP-β-CDs was studied by interacting with both molecules through NMR, as it provides the most direct evidence of the inclusion of the host molecule inside the CD [50]. By this technique, it has been possible to study the interaction mode of the HP-β-CD with different compounds, since if a host molecule is incorporated into the cavity of the HP-β-CD, the coupling constants of the protons from within the cavity of the HP-β-CDs (H_3_ and H_5_) undergo a modification; nevertheless, the protons of the external zone of the CDs do not present any change in their constants [51].

Table 3 shows the chemical shift values of carvacrol and HP-β-CD, both in their free state and when complexed in the methanol-d_4_ solution, as well as the differences between the signals of the free and complex molecules.

Changes in the chemical shifts of the free molecule can be seen relative to complexes, confirming the formation of the inclusion complexes. After the determination of the chemical shifts, a study was carried out using two-dimensional (2D) NMR spectroscopy. This technique is based on the existence of a very important effect relating to the determination of structures by NMR, the so-called Overhauser effect (NOE), which is due to an interaction between two protons that are closely located in space. This was observed in the spectra of NOESY or ROESY.

**Figure 7:**
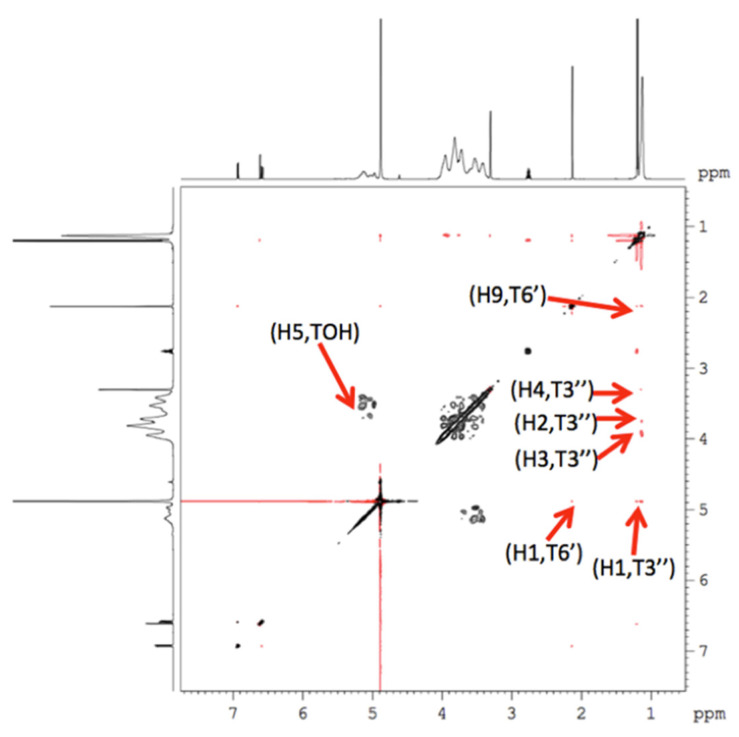
ROESY spectrum of the carvacrol-HP-β-CD complex in methanol-d_4_.

The presence of crossovers in the NOE between the protons of the two species indicates the presence of steric contacts within 0.4 nm. In order to obtain more conformational information, a 2D ROESY spectrum of the inclusion complex was created, as shown in Figure 7.

### 3.4. Molecular Docking Simulations

**Figure 8:**
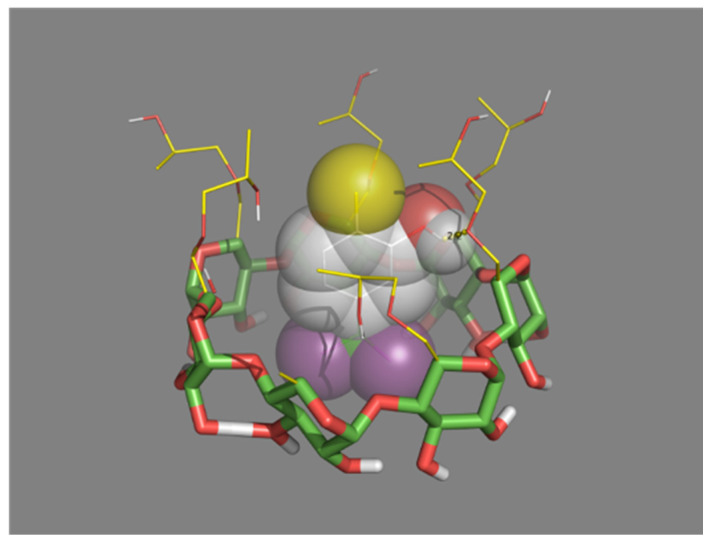
Structure of the carvacrol inclusion complex, with HP-β-CDs.

In order to understand how HP-β-CD interacts with carvacrol, coupling simulations were performed. The structural information of carvacrol, in combination with HP-β-CD, obtained by molecular docking is shown in Figure 8.

Carvacrol binds strongly to the inner cavity of HP-β-CDs. The hydrogen C3″ atoms from the isopropyl group in carvacrol interact (see Figure 2a) with the hydrogen atoms H1, H2, H3, and H4 from HP-β-CD (the lilac spheres in Figure 8), and the hydrogen from the hydroxyl group from carvacrol interacts with the hydrogen atoms of H5 from HP-β-CD (the red sphere from Figure 8). There is also an interaction between the C6′ proton of carvacrol with the H1 and H9 protons of HP-β-CDs. These results are in agreement with the 1H 2D-ROESY NMR data obtained, as shown in Figure 7. It is also clear that with this conformation, the carvacrol binds tightly into HP-β-CD’s hydrophobic core.

### 3.5. Differential Scanning Calorimetry (DSC) and Thermogravimetric Analysis (TG)

**Figure 9:**
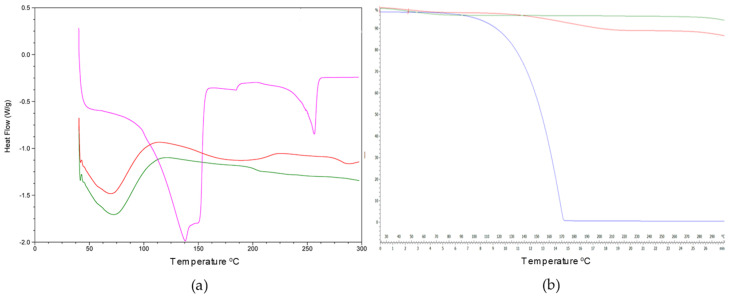
(**a**). Curves of the DSC carvacrol (pink line), MWI carvacrol-HP-β-CDs (red line), and HP-β-CDs (green line). (**b**). The TG curves of HP-β-CD (green line), MWI HP-β-CD/carvacrol (red line), and carvacrol (blue line).

The obtained thermograms for DSC and the thermogravimetric analysis are shown in Figure 9. The DSC curve of carvacrol has an endothermic band at around 140 °C. For HP-β-CD, due to its amorphous nature, a large endothermic peak was observed at approximately 70 °C (Figure 9a), associated with the loss of the water molecules, and a small variation was seen at 210 °C, due to the transformation of the molecule [52,53]. However, this signal was significantly reduced when the carvacrol was complexed into HP-β-CDs, suggesting a water repulsion process during complex formation (Figure 9a).

The DSC curve of the inclusion complex formed by carvacrol-HP-β-CD (Figure 9a) did not show the endothermic peaks that are characteristic of carvacrol (Figure 9a), indicating that this monoterpene was protected from thermal treatment due to the formation of an inclusion complex with HP-β-CD [54].

The TG analysis (Figure 9b) indicated that there was a mass loss at around 50 °C, corresponding to the water molecules inside the HP-β-CD. In addition, the TG curve of HP-β-CD shows a 5% weight loss; however, the curve of the complex formed by carvacrol and HP-β-CD has a weight loss of around 2.79%; this is due to the lower concentration of water molecules inside the CD since they have been displaced by carvacrol.

### 3.6. FTIR

**Figure 10:**
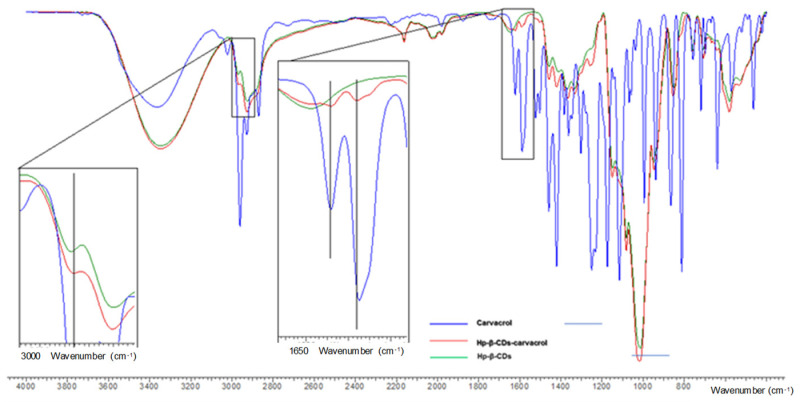
The FTIR spectrum of HP-β-CDs (**green line**), carvacrol-HP-β-CDs complexes by US (**red line**), and carvacrol (**blue line**). The vertical lines indicate the maximum of the HP-β-CDs curve.

The variations in the size, displacement, and intensity of the infrared absorption peaks of the host molecule provide sufficient information to confirm the formation of host inclusion complex 51. The infrared spectrum (IR) of HP-β-CD (Figure 10) shows several peaks: 3341 cm^−^^1^ (O-H voltage vibrations); 2923 cm^−^^1^ (C-H voltage vibrations); 1643 cm^−^^1^ (O-H flexural vibrations); 1157 cm^−^^1^ (C-O vibration); 1012 cm^−^^1^ (C-O-C stretching vibrations); 850 cm^−^^1^ (α-type glycosidic linkage); 2967 cm^−^^1^ (symmetrical anti-vibration of the methyl groups); 1375 cm^−^^1^ (bending methyl vibration).

In the IR spectrum of carvacrol (Figure 10), we can see the stretching bands associated with the -OH in the 3361 cm^−^^1^ region, the flexion bands out of the OH plane at 1457 cm^−^^1^, the stretching bands at the C=C aromatic compound at 1619 and 1587 cm^−^^1^, and the aromatic CH bond outside the 736 cm^−^^1^ plane. In addition, there are symmetric and asymmetric stretching bands of methyl appearing at 2869 and 2958 cm^−^^1^, respectively. Finally, a flexion band appears outside the plane of the C-H at 811 cm^−^^1^. In Figure 9, it can be noted that the bands of the free carvacrol molecules were covered by the peaks of the carvacrol-HP-β-CD complex because the amount of host molecules is not more than 10–15% (*w*/*w*) in the inclusion complexes [55].

In addition, it was observed that the stretching peaks of the C=C aromatic bond of carvacrol appear at 1619 cm^−^^1^ and 1587 cm^−^^1^. The first one maintains the wavelength when the inclusion complex of carvacrol with the HP-β-CD is formed, but the second undergoes displacement at 1589 cm^−^^1^. The peak corresponding to the asymmetric vibration of the methyl group of the hydroxypropyl moiety, appearing at 2967 cm^−^^1^, is also shifted to 2965 cm^−^^1^ once the inclusion complex has been formed. These slight changes between the free compounds and their complexed forms provide evidence for the interaction of carvacrol and HP-β-CDs.

### 3.7. The Cytotoxicity of Carvacrol and Its Complexes

Monoterpene phenols, such as carvacrol and thymol, have been shown to exert anti-proliferative effects on tumoral cells. However, the mechanisms of its anti-cancer activity are not yet elucidated. The cell proliferation of free carvacrol on tumoral and healthy cell lines was examined.

**Figure 11:**
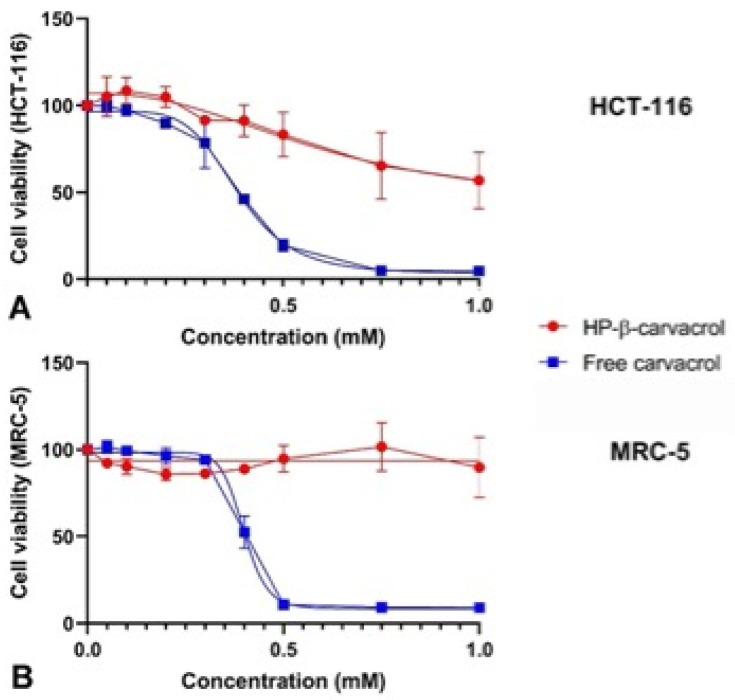
The dose-dependent effect of carvacrol and HP-β-CD-carvacrol complexes on (**A**) HCT-116 colorectal cancer cells and (**B**) MRC-5 lung fibroblast cells, with viability (72 h incubation). The data shown represent the averaged and SD values of the triplicates.

Cell viability studies demonstrated two different behaviors for the HCT-116 and MRC cell lines (Figure 11). The viability of the MRC-5 cell line was statistically higher in the presence of encapsulated carvacrol (IC_50_ 40.78 mM), when compared to the free form (IC_50_ 0.3982 mM) (*p* < 0.001). Besides, IC_50_ was calculated in the HCT-116 cells, suggesting that the cells tolerated a gradual delivery of the compound (IC_50_ 0.6104 mM) slightly better than in the free form (IC_50_ 0.3852 mM) (Figure 11) (linear regression, *p* < 0.001). Although cell viability was still encouragingly high at 1 mM for HCT-116 (∼60% of viable cells), it was significantly higher in the MRC-5 cells (∼80%), as denoted by both IC_50_ values (*p* < 0.001).

**Figure 12:**
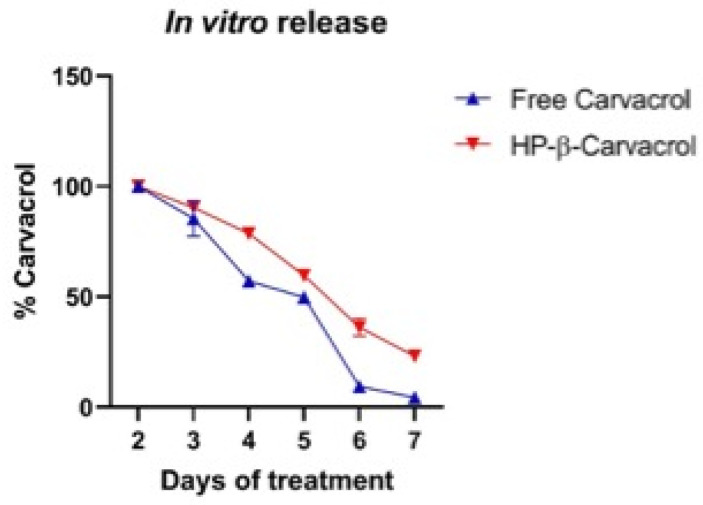
The release of carvacrol, both free and complexed, with HP-β-CD in the presence of HCT-116 cells, at 37 °C, after 7 days of treatment, where n = 3.

The HP-β-carvacrol complex and free carvacrol release were evaluated in order to better understand the viability results. The complex seemed to dissociate carvacrol from the cavity in a sustained manner when compared to carvacrol in solution. Figure 12 showed a soluble drug of only 4.37 ± 1.61% at the end of the 7-day period for free carvacrol, compared to the enhanced solubility of the optimized complex at 23.18 ± 1.17% in dissolution. In accordance with our results, complexes prepared using HP-β-CD showed better drug release, which could be attributed to its enhanced solubility and the resultant improved dissolution [56].

## 4. Conclusions

Carvacrol can be complexed into CDs, increasing its aqueous solubility by forming 1:1 complexes. In addition, it has been found that the formation of carvacrol inclusion complexes with CDs varies according to the pH of the medium, obtaining better K_C_ values at a neutral pH. The highest K_C_ value was obtained with HP-β-CDs, followed by α-CDs and β-CDs. Despite the fact that two methods of preparation of solid complexes with carvacrol with HP-β-CDs have been evaluated, the results obtained for K_C_, encapsulation efficiency, active matter loading, and complex stability support the use of the MWI in the preparation of solid complexes of carvacrol.

The results obtained by nuclear magnetic resonance and molecular coupling confirm carvacrol entrapment into the cavity of HP-β-CDs. In addition, the determinations carried out by thermogravimetry, differential scanning calorimetry, and Fourier transform infrared spectrometry on isolated reactants and their respective complexes confirm that, in all cases, complex formation between the monoterpene under study and HP-β-CDs is effective.

Furthermore, it has been evidenced that HP-β-CD-carvacrol complexes show better antitumor activity than free carvacrol since the HP-β-CD-carvacrol complexes are capable of inhibiting the growth of the tumor cell line, HCT-116 (43%), slightly affecting the viability of the healthy cell line, MRC-5, which was in the order of 11%.

## Figures and Tables

**Table 1:** Solubility, K_C_, and complexation efficiency between carvacrol and the various types of CDs at different pHs.

Cyclodextrins	pH	S_0_ (mmol L^−1^)	K_C_ (L mol^−1^)	CE	MR
α-CDs	3.5	2.76 ± 0.20	108 ± 16	0.298	1:4
	5.5	1.65 ± 0.16	192 ± 24	0.317	1:4
	6.5	0.92 ± 0.11	239 ± 42	0.220	1:5
	7.0	0.56 ± 0.03	400 ± 56	0.224	1:5
	8.5	0.76 ± 0.13	284 ± 37	0.216	1:5
β-CDs	3.5	2.76 ± 0.20	140 ± 21	0.386	1:3
	5.5	1.65 ± 0.16	748 ± 19	1.234	1:1
	6.5	0.92 ± 0.11	866 ± 180	0.797	1:2
	7.0	0.56 ± 0.03	3466 ± 115	1.941	1:1
	8.5	0.76 ± 0.13	580 ± 45	0.441	1:3
HP-β-CDs	3.5	2.76 ± 0.20	198 ± 19	0.546	1:2
	5.5	1.65 ± 0.16	327 ± 37	0.540	1:2
	6.5	0.92 ± 0.11	1521 ± 73	1.399	1:1
	7.0	0.56 ± 0.03	5042 ± 176	2.824	1:1
	8.5	0.76 ± 0.13	973 ± 94	0.739	1:2

MR: molar ratio. Values are the means of three determinations.

**Table 2:** Encapsulation efficiency (EE) and drug-loading (DL).

HP-β-CDs	MWI		US	
	EE (g/100 g)	DL (g/100 g)	EE (g/100 g)	DL (g/100 g)
20 mM	51.96 ± 5	4.97 ± 0.04	50.60 ± 4	3.06 ± 0.04
30 mM	54.90 ± 3	4.33 ± 0.05	50.86 ± 3	3.29 ± 0.05
50 mM	66.37 ± 3	6.48 ± 0.07	64.55 ± 3	3.64 ± 0.08
75 mM	55.31 ± 3	5.27 ± 0.08	54.50 ± 4	3.35 ± 0.03
100 mM	59.65 ± 3	4.34 ± 0.04	55.71 ± 2	2.90 ± 0.06

**Table 3:** Chemical displacement of carvacrol and HP-β-CD, both in free form and when complexed in methanol-d4.

Molecules	H-Atom	δ/ppm (Free)	δ/ppm (Complex)	Δδ (Complex-Free)/ppm
Carvacrol	H-C (5)	6.566	6.577	−0.011
	H-C (4)	6.619	6.611	0.008
	H-C (2)	6.917	6.929	−0.012
	H-C (3′)	2.74	2.758	−0.018
	H-C (6′)	2.126	2.124	0.002
	H-C (3″)	1.176	1.195	−0.019
HP-β-CDs	H-C (1)	5.074	5.121	−0.047
	H-C (2)	3.723	3.73	−0.007
	H-C (3)	3.947	3.952	−0.005
	H-C (4)	3.418	3.417	0.001
	H-C (5)	3.534	3.533	0.001
	H-C (6)	3.821	3.815	0.006
	H-C (9)	1.126	1.125	0.001

## Data Availability

Not applicable.
